# Forms of aggression, peer relationships, and relational victimization among Chinese adolescent girls and boys: roles of prosocial behavior

**DOI:** 10.3389/fpsyg.2015.01264

**Published:** 2015-08-21

**Authors:** Shujun Wang, Wei Zhang, Dongping Li, Chengfu Yu, Shuangju Zhen, Shihua Huang

**Affiliations:** ^1^School of Psychology & Center for Studies of Psychological Application, South China Normal UniversityGuangzhou, China; ^2^School of Psychology, Central China Normal UniversityWuhan, China; ^3^School of Economic and Management, Guangzhou University of Chinese MedicineGuangzhou, China

**Keywords:** relational aggression, overt aggression, relational victimization, peer victimization, peer relationship, peer rejection, prosocial behavior, gender difference

## Abstract

Through a sample of 686 Chinese adolescents (mean age = 13.73 years; 50% girls), we examined the compensatory and moderating effects of prosocial behavior on the direct and indirect associations between forms of aggression and relational victimization mediated by peer relationships among adolescent girls and boys. The results indicated that only adolescent girls’ relationally aggressive behaviors could be directly linked with their experiences of relational victimization, and both relationally and overtly aggressive adolescent boys and girls might be more often rejected by their peers, which, in turn, could make them targets of relational aggression. Next, we found that prosocial behavior indirectly counteracts the effects of aggression on relational victimization through reducing adolescents’ peer rejection and promoting adolescents’ peer attachment. In addition, relationally aggressive girls with high levels of prosocial behavior might be less rejected by peers; however, they might also have lower levels of peer attachment and be more likely to experience relational victimization. Last, adolescent boys scored higher on risks, but lower on the protective factors of relational victimization than girls, which, to some degree, might explain the gender difference in relational victimization. Finally, we discussed the theoretical and practical implications of these findings.

## Introduction

After attracting significant attention from researchers in the late 1980s, peer victimization has been consistently identified as a common problem related to both internalizing (see Reijntjes et al., 2010) and externalizing (see Reijntjes et al., 2011) problems as well as other negative developmental outcomes (e.g., Seeley et al., 2009) across countries.

In addition to the overt form of peer victimization (e.g., Olweus, 1978), the significance of an empirical focus on relational victimization has been recognized and strongly emphasized (Crick et al., 2001). This sub-type of peer victimization involves being the targets of peers’ relational aggression, a type of behavior aimed at harming others by intentionally damaging or manipulating their interpersonal relations or by threatening to destroy these relations (Crick et al., 2001). Nowadays, the increasing number of studies has evidenced that the experience of such peer maltreatment might also have negative impacts on victims (e.g., Crick and Nelson, 2002; Dempsey and Storch, 2008; Rudolph et al., 2009). More importantly, a study of relational victimization might provide a better understanding of girls’ social development (Crick and Grotpeter, 1996) since, unlike the studies of overt victimization indicating that the victims were mainly boys, studies of relational victimization have found that the phenomenon was prevalent among both genders (e.g., Crick and Nelson, 2002; Putallaz et al., 2007).

Therefore, in response to Crick et al.’s (2002) appeal for more studies on antecedents of relational victimization, the present study explored how adolescents’ social behaviors (i.e., aggressive and prosocial behaviors) and peer relationships are correlated to their possibility of experiencing relational victimization.

### Forms of Aggression, Peer Relationships, and Relational Victimization

Studies of both Western and Chinese samples have found that compared to passive victims and non-victimized children, aggressive victims, a subgroup of victimized children who are oppositional and aggressive, appeared to represent an extreme group that is at the greatest risk for negative peer group outcomes and psycho-social adjustment problems (Xu et al., 2003). These children are harassed often because their aggressive behaviors irritate peers. Concerning the relationship between aggression and victimization, we drew from the theoretical social process model of the causes of peer harassment proposed by Boivin et al. (2001). According to the model, aggression and victimization may be linked in two pathways. First, aggression may directly lead to peer victimization (Boivin et al., 2001). For this part of the model, a number of studies have offered empirical support (e.g., Crick et al., 1999; Kochenderfer-Ladd, 2003; Sullivan et al., 2006). Second, aggression may indirectly lead to peer victimization mediated by negative peer status (i.e., peer rejection). Although, comparatively, the indirect pathway has not been adequately tested, the different parts of this model have been found to be interlinked. For example, studies have found that relational and physical aggression predicted peer rejection (e.g., Crick et al., 2006; Schwartz et al., 2010; Tseng et al., 2013) in both Chinese and Western cultures, and that peer rejection was related with relational and physical victimization (e.g., Crick et al., 1999). Ostrov (2008) conducted a short-term longitudinal study of an early childhood sample to systematically explore the two pathways from the subtypes of aggression to peer victimization. Prospective findings have indicated that physical aggression predicted increases in relational victimization, but relational aggression predicted increases in relational victimization for only girls. Moreover, peer rejection mediated the associations between forms of aggression and relational victimization (Ostrov, 2008).

However, so far, no study has synthetically tested the two pathways among a sample of adolescents or in the background of Chinese culture. Given that relationally aggressive behaviors tend to increase during the early to middle years of high school (Underwood et al., 2009), in this study, we focused on adolescents. More importantly, as the Chinese culture emphasizes self-control, interdependence, and harmonious interpersonal relationships, dysregulated individual behaviors that damage these cultural values are likely to be harshly evaluated or punished (Chen and French, 2008). Accordingly, Chinese children who are aggressive may be more likely to be rejected by peers, and in turn, may be more likely to be victimized.

Moreover, the aforementioned Ostrov’s (2008) study was conducted without considering the role of friendship, the other important dimension of peer relationships (Bukowski and Hoza, 1989), which becomes an increased focus since adolescence (Rubin et al., 1998). Especially, in the relationship-oriented Chinese culture, given the high emphasis on interpersonal relationships, friendship plays a central role in the socialization of children (Chen, 2000). Accordingly, it may be the case that Chinese children who are aggressive tend to have fewer friends and lower quality of friendship, and thus might be more likely to be victimized. Actually, it has been evidenced that friendship, in both quantity and quality, was, to some extent, associated with aggression and victimization (e.g., Hodges et al., 1999; Scholte et al., 2009; Wang et al., 2009; Kawabata et al., 2010).

Therefore, based on the social process model of Boivin et al. (2001) as well as existing studies, we first examined the direct and indirect pathways from overt and relational aggression to relational victimization through three aspects of peer relationships (i.e., peer rejection, friendship quantity, and friendship quality) in a Chinese adolescent sample.

### Models of Resilience: Roles of Prosocial Behavior

Researchers have identified several important models of resilience to explain how promotive factors change the trajectory from risks to negative outcomes. For instance, a compensatory model is defined when a promotive factor counteracts the effects of a risk factor by linking with the outcome in the opposite direction of the risk factor (see Fergus and Zimmerman, 2005). Previous research has documented a negative link between prosocial behavior and peer victimization (e.g., Schwartz et al., 1993; Boulton and Smith, 1994; Ostrov and Keating, 2004), thus, suggesting that it might serve as a compensatory factor that counteracts the effects of risk factors, such as aggressive behavior, on peer victimization. With the addition of the documented links between prosocial behavior and peer relationships (e.g., Asher and Coie, 1990; Zimmer-Gembeck et al., 2005), we assumed that prosocial behavior is a compensatory factor that can counteract the effects of aggression by linking with relational victimization in the opposite direction both directly and indirectly through peer relationships.

In contrast, a protective model, another important model of resilience, is defined when a promotive factor moderates or reduces the effects of a risk on a negative outcome (see Fergus and Zimmerman, 2005). According to Hawley’s resource control theory (RCT; Hawley, 1999), which focuses on the function of behavior (i.e., resource control) rather than the structure of behavior (i.e., form), resource control in a peer group can be achieved via two broad strategies: coercive and prosocial. Generally, coercive controllers are often aggressive while prosocial controllers tend to attain their goals via prosocial behaviors, and bistrategic controllers have characteristics in common with both coercive and prosocial controllers. This theory proposes that bistrategic individuals (i.e., aggressive individuals who also display prosocial behaviors) should be socially successful, achieving and maintaining high social dominance status in a peer group (see Hawley, 2007), and thus suggests that prosocial behaviors may reduce the effects of aggression on adjustment problems. Actually, there has been evidence that prosocial behaviors might play a role as a moderator in the relationship between aggression and peer status (e.g., Cillessen and Mayeux, 2004) as well as that between aggression and friendship quality (e.g., Sebanc, 2003). However, the specific protective mechanisms of prosocial behavior in both pathways from forms of aggression to relational victimization remain unclear, and therefore we focused on such mechanisms.

### Gender Difference

Although the majority of studies on relational victimization have involved the issue of gender, gender difference in children’s experiences of relational victimization has remained controversial. Many researchers have maintained that girls experience more relational victimization than boys (e.g., Crick and Nelson, 2002; Putallaz et al., 2007; Smith et al., 2010; Farisa and Felmlee, 2014), in accordance with the hypothesis of gender specificity (Crick and Grotpeter, 1995) and the evolutional hypothesis (see Côté, 2007). However, the influence of gender on relational victimization might be more complicated. For example, many studies have argued that such gender difference might be negligible (e.g., Storch et al., 2005; Vuijk et al., 2007; Gianluca, 2008; Sentse et al., 2013). Moreover, an increasing number of studies have provided evidence for more relational victimization among boys rather than girls (e.g., Ji et al., 2004; Khoury-Kassabri et al., 2004; Schwartz et al., 2010), including those conducted with Chinese samples. For instance, Ji et al. (2004) examined Chinese and British primary and secondary school children to explore the cultural variations of gender difference in the prevalence of bullying and victimization. Their findings showed that Chinese boys from both primary and secondary schools reported more experience of indirect bullying than girls, whereas in Britain, the contrary was the case. Moreover, researchers also found some evidence that, in the Chinese cultural context, peer victimization is a more central issue for boys than girls (i.e., boys are initiators and recipients of bullying more often than girls; Schwartz et al., 2001). Due to these potential cultural differences, we expect a significant gender difference in relational victimization.

### The Present Study

The main goal of the present study was to test a mediation model and a moderated mediation model, exploring the relationship between forms of aggression and relational victimization when considering the roles of peer relationships and prosocial behavior among adolescent girls and boys, respectively. Based on our literature review, we propose the following hypotheses:

*Hypothesis 1:* Both relational and overt aggression are directly and indirectly linked to relational victimization, which is partially mediated by all aspects of peer relationships.
*Hypothesis 2a:* Prosocial behavior is a compensatory factor that can counteract the effects of aggression by linking with relational victimization in the opposite direction both directly and indirectly through peer relationships.
*Hypothesis 2b:* Prosocial behavior moderates both direct and indirect pathways from forms of aggression to relational victimization. However, due to the lack of evidence to date, we did not have a specific hypothesis regarding the moderating effect of prosocial behavior on specific pathways.
*Hypothesis 3:* Adolescent boys report more experience of relational aggression than adolescent girls.

## Materials and Methods

### Participants

Participants were 703 students (49% girls) in grades 7–9 (11–16 years of age; mean age = 13.73, SD = 1.10) from four public middle schools in Guangzhou area, southern China. The proportions of each grade were 35, 32, and 33%, respectively. Totally, 47% of the fathers and 61% of the mothers had an educational level of high school or below. Seven adolescents did not write down their gender information and another 10 failed to answer most of the items. The data of these 17 adolescents were considered as invalid and were thus eliminated. Finally, the data of the 686 adolescents (50% girls) were included in the following analyses.

### Measures

#### Relational Victimization

Adolescents’ relational victimization was judged using the Social Experience Questionnaire-Self Report (SEQ-S) developed by Crick and Grotpeter (1996). The questionnaire, which assesses overt and relational victimization, and prosocial behaviors from peers, has been widely used in existing research. Studies aiming at psychometric evaluation of the questionnaire have reported favorable internal consistency reliabilities (Cronbach’s α), factorial validity (Storch et al., 2005; Desjardins et al., 2013) and test–retest stability (Storch et al., 2005). The students were asked to rate each item on a five-point scale ranging from 1 (never happened) to 5 (always happened), thus indicating the frequency of occurrence. Examples of items in the Relational Victimization Sub-scale included: “How often does a classmate tell lies about you to make other kids not like you anymore?” and “How often does a kid try to keep others from liking you by saying mean things about you?” In the current sample, the internal consistency reliability (Cronbach’s α) for relational victimization (α = 0.80) was favorable. In addition, confirmatory factor analysis (CFA), using AMOS 17.0 (Arbuckle, 2008), was utilized to examine the structure of relational victimization. The five-item structure of relational victimization proposed by Crick and Grotpeter (1996) was used as the basis for comparison. Results from the CFA suggested that the structure was fit well to the sample data (χ^2^/*df* = 1.30, CFI = 1.00, TLI = 1.00, IFI = 1.00, RMSEA = 0.021). Standardized factor loadings of each item were significant at the 0.001 level, with the coefficients ranging from 0.48 to 0.83.

#### Aggressive and Prosocial Behavior

A total of 14 items were selected from a modified version of Crick and Grotpeter’s (1995) peer-nomination measure (Dempsey et al., 2006) to assess relational aggression (five items, e.g., “Find the names of three kids who tell their friends that they will stop liking them unless the friends do what they say.”), overt aggression (five items, e.g., “Find the names of three kids who hit, kick, or punch other kids at school.”) and prosocial behavior (four items, e.g., “Find the names of three kids who help out others when they need it.”). With a class roster that included the class members’ names and identification numbers next to each name, the peer nomination materials were handed out to all of the participants. Then, the participants nominated up to three classmates for each question. The nominations were computed by forms of aggression and prosocial behavior, and then standardized within each classroom. This process resulted in a *z*-score for each participant of which the scores above zero suggested higher levels of aggression and prosocial behavior.

#### Peer Relationships

We assessed three aspects of peer relationships belonging to two dimensions: peer status and friendship (Bukowski and Hoza, 1989). The first variable, *peer rejection*, which represents negative peer status, was assessed with one item (i.e., “Find the names of three kids you like to play with or do activities with the least”) from the aforementioned version of Crick and Grotpeter’s (1995) peer-nomination measure. The participants nominated three classmates that they liked the least in their classroom and these “least liked” nominations were computed and standardized within each classroom, thus resulting in a *z*-score for each participant of which the scores above zero suggested higher levels of peer rejection. Peer nomination has been shown to be reliable and valid procedures to measure social status in previous studies (Terry, 2000; Cillessen and Borch, 2006). The second variable, *friendship quantity or number of friends*, was assessed using one question developed for the present study, “How many friends do you have in the school?” The participants were asked to rate on a four-point scale ranging from 0 to 3 (0 = no friend, 1 = one friend, 2 = two to three friends, 3 = more than four friends).

Finally, the present study assessed *friendship quality* using the simple version of the Inventory of Parent and Peer Attachment (IPPA) developed by Armsden and Greenberg (1987) and revised by Raja et al. (1992). The IPPA was developed to assess adolescents’ perceptions of positive or negative dimensions of relationships with their parents and close friends. Three broad dimensions (i.e., quality of communication, degree of mutual trust, and extent of alienation) were assessed, each including four items for parents and peers separately. The instrument has been proved applicable with acceptable reliability in studies with adolescents (Raja et al., 1992). In the present study, only the peer attachment scale was used. The participants were asked to rate each item on a five-point scale ranging from 1 (totally disagree) to 5 (totally agree). The examples included: “My friends encourage me to talk about my difficulties,” “My friends listen to what I have to say,” and “I get upset a lot more than my friends know about.” Cronbach’s α coefficient for the whole measure was 0.82 in our study, and that for the Communication, Trust and Alienation Sub-scales were 0.83, 0.60 and 0.74.

To sum up, the present study examined five observable variables (i.e., relational aggression, overt aggression, prosocial behavior, peer rejection and number of friends) and two latent variables (i.e., relational victimization and peer attachment). As presented in **Table 2**, the indicators of relational victimization were the five items in the scale; and the three indicators of peer attachment were average scores of items in the Communication, Trust and Alienation Sub-scales.

### Procedure

All materials and procedures were approved by the Ethics in Human Research Committee of the authors’ University. Since the participants of the study are Chinese adolescents, all measures were administered in Chinese. Hence, before the survey was conducted, all the English versions of questionnaires and peer nomination materials were translated into Chinese. This process included five steps. In Step 1, four graduate students from the psychology department independently completed the translation and then held discussions to form a unique Chinese version. In Step 2, a graduate student from the English department back-translated the Chinese version into English. In Step 3, the five graduate students discussed with the first author to check the back-translated version against the original version to ensure no distortion in the meaning had been made. In Step 4, the questionnaires (as a pilot study) were administered to 90 students (30 students from each grade level). Finally, in Step 5, based on their feedback, some vague expressions were clarified after which we employed the final Chinese version for the analyses.

We administered a 20-min survey to the participants after obtaining informed consents from the school, the parents and the students. The trained research assistants explained all the requirements of the survey by reading the standardized instructions aloud. They also emphasized that the students should answer all the questions honestly and independently. All of the participants were assured of their confidentiality in regard to the information collected, and they were requested to not discuss their individual responses with any of their peers, as to not “hurt anyone’s feelings.” Additionally, according to Dempsey et al. (2006), we conducted a brief discussion immediately after the questionnaires had been completed, in which the participants were encouraged to give examples regarding the potential consequences of talking about their responses with their peers (e.g., hurt feelings, friendship problems).

## Results

### Descriptive Statistics

The findings of descriptive statistics indicated that approximately 62% of the boys and 60% of the girls had previously been the targets of peers’ relational aggression. In addition, roughly 3.8% of the boys and 2.0% of the girls reported “often” in regard to having such an experience. The means, standard deviations, and gender differences for all variables as well as the Pearson correlation coefficients among all variables for both the adolescent boys and girls are presented in **Table 1**. Gender difference was examined using the analysis of variance (ANOVA) procedures. Four variables showed significant gender differences. Compared with adolescent girls, adolescent boys had higher scores on relational victimization, relational aggression, and overt aggression, but lower scores on peer attachment. Thus, we obtained support for Hypothesis 3, which predicted that adolescent boys would report more experience of relational aggression than adolescent girls.

**Table 1:** Means, SD, and Pearson correlation coefficients between all variables for girls and boys.

Variables	Girls (*n* = 343)		Boys (*n* = 343)			1	2	3	4	5	6	7
	*M*	SD	*M*	SD	F Test							
(1) Relational Victimization	1.76	0.64	1.99	0.74	4.24∗	1	0.23∗∗	0.09	-0.04	0.20∗∗	-0.08	-0.19∗∗
(2) Relational aggression	-0.16	0.80	0.13	1.07	16.23^∗∗∗^	0.13∗	1	0.65∗∗	-0.10	0.66∗∗	-0.02	-0.04
(3) Overt aggression	-0.31	0.49	0.28	1.14	76.87^∗∗∗^	0.06	0.73∗∗	1	-15∗∗	0.55∗∗	0.07	-0.02
(4) Prosocial behavior	0.03	1.01	-0.03	1.00	0.51	-0.08	-0.01	-0.03	1	-0.21∗∗	0.03	0.16∗∗
(5) Peer rejection	0.01	1.03	-0.01	0.97	0.05	0.18∗∗	0.58∗∗	0.55∗∗	-0.22∗∗	1	-0.09	-0.03
(6) Number of friends	2.72	0.56	2.80	0.51	3.14	-0.22∗∗	0.01	0.05	0.08	0.05	1	0.23∗∗
(7) Peer attachment	3.88	0.64	3.66	0.70	19.67^∗∗∗^	-0.25∗∗	-0.10	-0.06	0.12∗	-0.09	0.24∗∗	1

*Girls’ correlations were presented above the diagonal and boys’ correlations below the diagonal. ∗*p* < 0.05, ∗∗*p* < 0.01, ^∗∗∗^*p* < 0.001.*

**Table 2:** Observable indicators of *latent variables.*

Latent variables	Standardized loading coefficients
**Relational victimization**	
(1) How often do other kids leave you out on purpose when it is time to play or do an activity?	0.48^∗∗∗^
(2) How often does a kid who is mad at you try to get back at you by not letting you be in their group anymore?	0.58^∗∗∗^
(3) How often does a classmate tell lies about you to make other kids not like you anymore?	0.83^∗∗∗^
(4) How often does another kid say they won’t like you unless you do what they want you to do?	0.58^∗∗∗^
(5) How often does a kid try to keep others from liking you by saying mean things about you?	0.79^∗∗∗^
**Peer attachment**	
(1) Communication	0.63^∗∗∗^
(2) Trust	0.90^∗∗∗^
(3) Alienation	0.36^∗∗∗^

*^∗∗∗^p < 0.001.*

### Mediation

Using the AMOS 17.0 (Arbuckle, 2008), we first tested Hypothesis 1 and 2a in a mediation model for adolescent girls and boys, respectively. Hypothesis 1 predicted that peer relationships would partially mediate the associations between forms of aggression and relational victimization, and Hypothesis 2a predicted that prosocial behavior might play a compensatory role partially through peer relationships. The SEM results indicated that both models of boys and girls fit the data quite well (Model 1 for girls: χ^2^/*df* = 2.56, CFI = 0.95, TLI = 0.91, IFI = 0.95, RMSEA = 0.068; Model 2 for boys: χ^2^/*df* = 1.17, CFI = 0.99, TLI = 0.99, IFI = 0.99, RMSEA = 0.023).

#### Peer Relationships as Mediators

**FIGURE 1:**
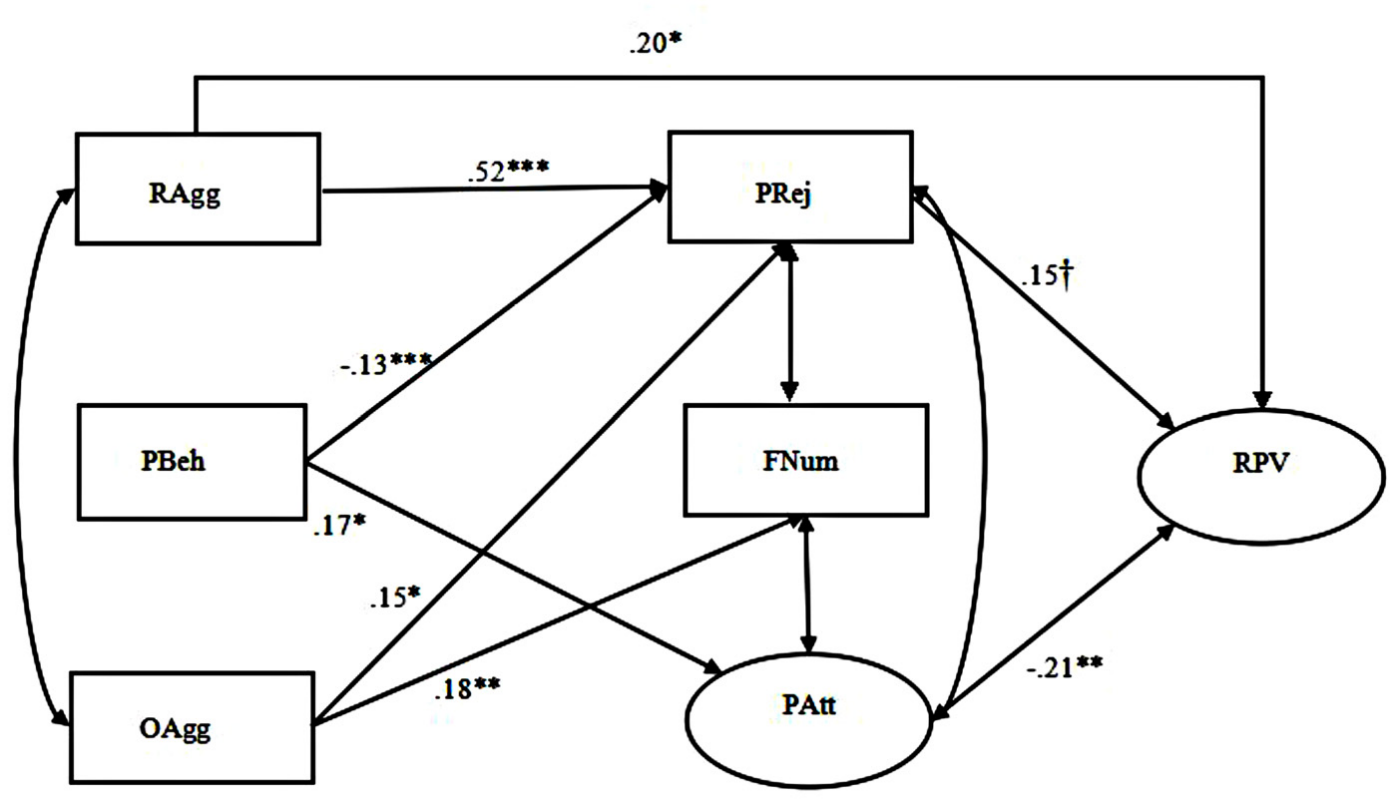
**Prosocial behavior compensates for the effects of relational and overt aggression on relational victimization mediated by peer relationships among adolescent girls (Model 1).** The latent variables were depicted by ellipses, and the observable variables were depicted by rectangles. Standardized coefficients were provided. Pathways that were non-significant were not shown for clarity. RAgg, relational aggression, PBeh, prosocial behavior, OAgg, overt aggression, PRej, peer rejection, FNum, friend number, PAtt, peer attachment, RPV, relational victimization. ^†^*p* = 0.07, ∗*p* ≤ 0.05, ∗∗*p* ≤ 0.01, ^∗∗∗^*p* ≤ 0.001.

**FIGURE 2:**
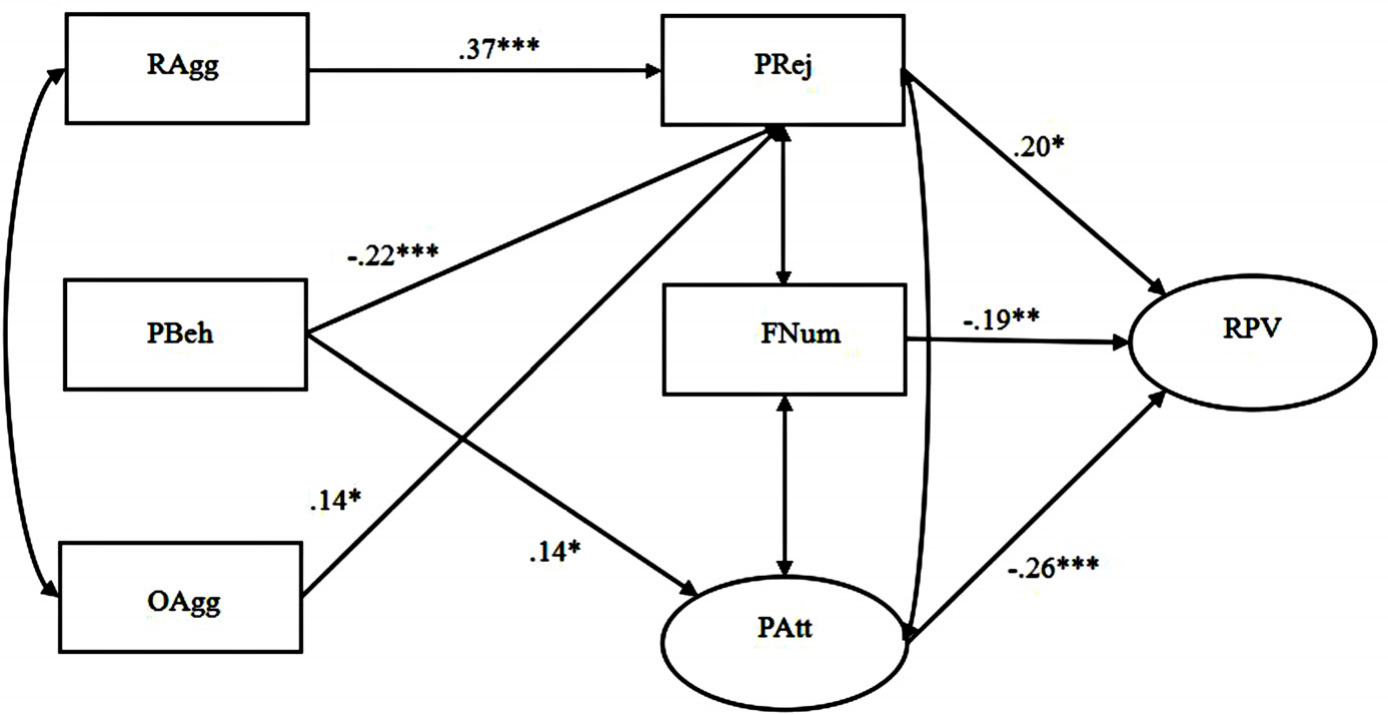
**Prosocial behavior compensates for the effects of relational and overt aggression on relational victimization mediated by peer relationships among adolescent boys (Model 2).** The latent variables were depicted by ellipses, and the observable variables were depicted by rectangles. Standardized coefficients were provided. Pathways that were nonsignificant were not shown for clarity. RAgg, relational aggression, PBeh, prosocial behavior, OAgg, overt aggression, PRej, peer rejection, FNum, friend number, PAtt, peer attachment, RPV, relational victimization. ∗*p* ≤ 0.05, ∗∗*p* ≤ 0.01, ^∗∗∗^*p* ≤ 0.001.

The path details of Model 1 and Model 2 are presented in **Figures 1** and **2**. For adolescent girls, relational aggression was both directly and indirectly associated with relational victimization partially mediated by peer rejection, whereas only the indirect link was found between overt aggression and relational victimization through peer rejection. For adolescent boys, both relational and overt aggression were found to indirectly correlate with relational victimization totally mediated by peer rejection. However, we failed to find any significant mediating effects of the other two aspects of peer relationships (i.e., number of friends and peer attachment) for neither the boys nor the girls. Although these findings suggested that our Hypothesis 1 is only partially supported, it is worth noting that peer attachment was negatively linked with relational victimization for girls, and both the number of friends and peer attachment were negatively associated with relational victimization for boys.

#### Prosocial Behavior as a Compensatory Factor

We examined the compensatory effect of prosocial behavior on both pathways from forms of aggression to relational victimization in the same mediation models (see **Figures 1** and **2**). The results showed that after controlling for the effects of relational and overt aggression, prosocial behavior indirectly reduced relational victimization by avoiding peer rejection and promoting peer attachment for both adolescent girls (Model 1) and boys (Model 2), thus suggesting that it might play a compensatory role in the negative links from forms of aggression to relational victimization for both genders. However, we failed to find a direct link between prosocial behavior and relational victimization among both the adolescent girls and boys. Therefore, our Hypothesis 2a has also been partially supported.

### Moderated Mediation: Prosocial Behavior as a Moderator

**FIGURE 3:**
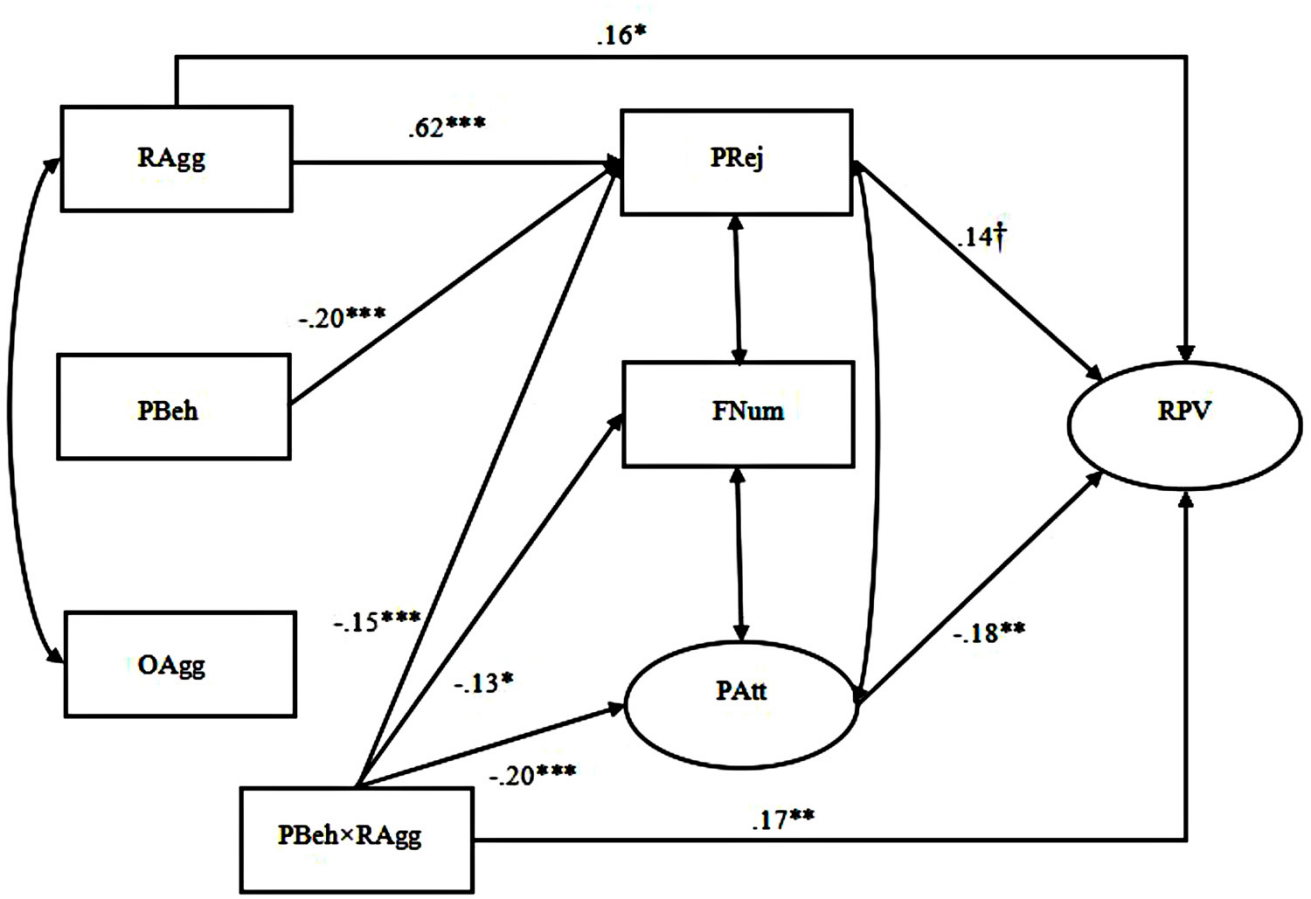
**Prosocial behavior moderates the effects of relational and overt aggression on relational victimization mediated by peer relationships among adolescent girls (Model 3.1).** The latent variables were depicted by ellipses, and the observable variables were depicted by rectangles. Standardized coefficients were provided. RAgg, relational aggression, PBeh, prosocial behavior, OAgg, overt aggression, PRej, peer rejection, FNum, friend number, PAtt, peer attachment, RPV, relational victimization. ^†^*p* = 0.07, ∗*p* ≤ 0.05, ∗∗*p* ≤ 0.01, ^∗∗∗^*p* ≤ 0.001.

**FIGURE 4:**
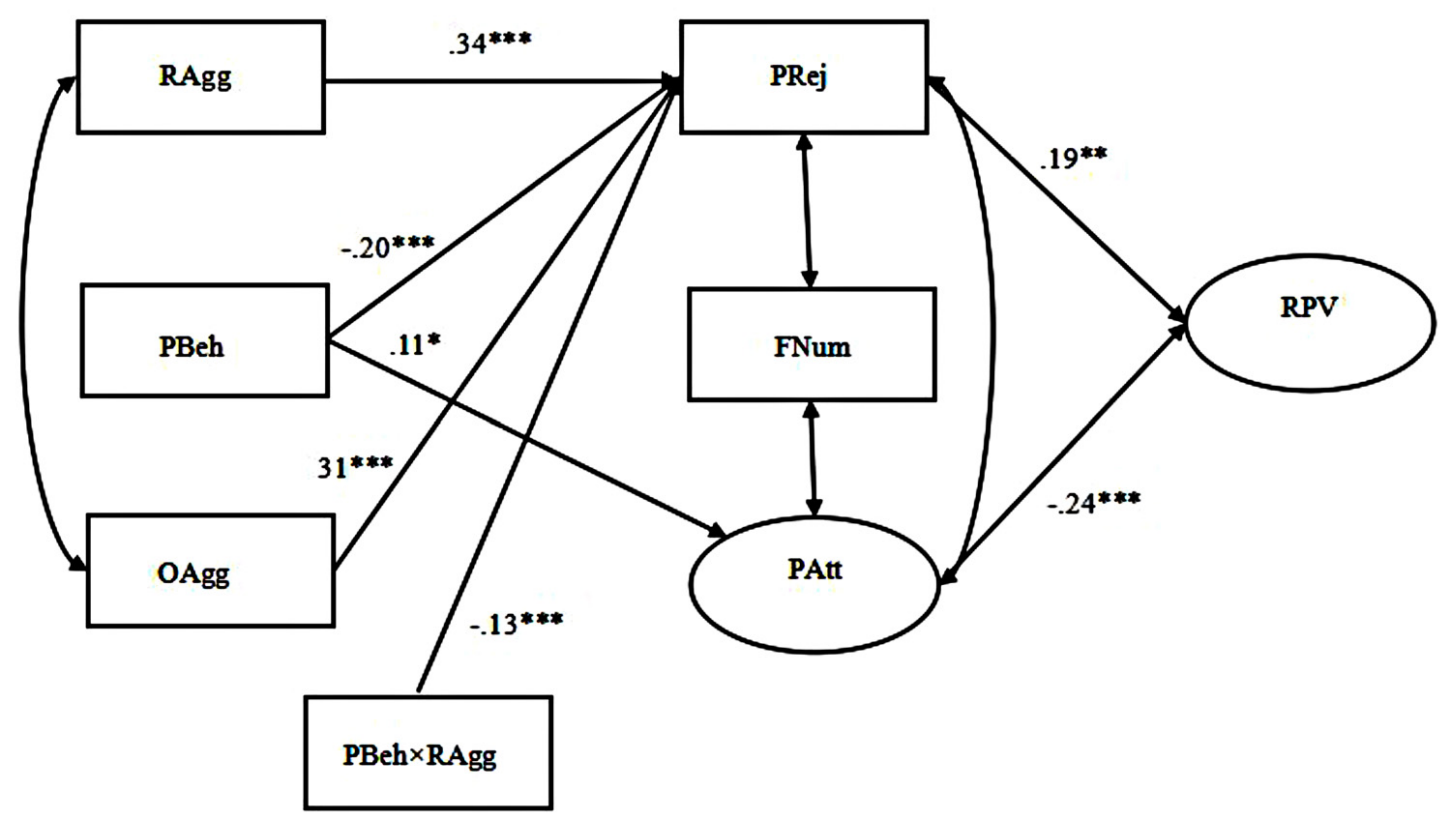
**Prosocial behavior moderates the effects of relational and overt aggression on relational victimization mediated by peer relationships among adolescent boys (Model 4.1).** The latent variables were depicted by ellipses, and the observable variables were depicted by rectangles. Standardized coefficients were provided. RAgg, relational aggression, PBeh, prosocial behavior, OAgg, overt aggression, PRej, peer rejection, FNum, friend number, PAtt, peer attachment, RPV, relational victimization. ∗*p* ≤ 0.05, ∗∗*p* ≤ 0.01, ^∗∗∗^*p* ≤ 0.001.

Importantly, we tested a moderated mediation model to synthetically examine Hypothesis 2b, which predicted that prosocial behavior would have moderating effects on both the direct (i.e., relational/overt aggression → relational victimization) and indirect (i.e., relational/overt aggression → peer relationships → relational victimization) pathways among the adolescent boys and girls, respectively. Moderated mediation, also known as conditional indirect effect, occurs when the mediating effect of a variable on an outcome variable depends on levels of another variable (Preacher et al., 2007). We tested this model following the suggestions of Aiken and West (1991). First, all independent variables (i.e., relational/overt aggression, prosocial behavior, peer rejection, number of friends, and peer attachment) were mean centered to better explain the moderating effects and reduce multicollinearity among variables. Then, five interaction terms (i.e., prosocial behavior × relational aggression, prosocial behavior × overt aggression, prosocial behavior × peer rejection, prosocial behavior × number of friends, and prosocial behavior × peer attachment) were added to establish Model 3 for the girls and Model 4 for the boys. Although the results suggested that both models adequately fit the data (Model 3: χ^2^/*df* = 3.01, CFI = 0.95, TLI = 0.90, IFI = 0.95, RMSEA = 0.077; Model 4: χ^2^/*df* = 1.30, CFI = 0.99, TLI = 0.98, IFI = 0.99, RMSEA = 0.029), we still chose to simplify both models by deleting all the non-significant paths and establish Model 3.1 for the girls (see **Figure 3**) and Model 4.1 for the boys (see **Figure 4**). The results of the model comparisons, as presented in **Table 3**, indicated that the fit statistics of Model 3.1 and Model 4.1 were better than those of Model 3 and Model 4. Therefore, we selected the more simplified models (i.e., Model 3.1 and Model 4.1).

**Table 3:** Model fit summary.

Model	χ^2^/*df*	CFI	TLI	IFI	RMSEA	χ^2^	*df*	Δ*df*	Δχ^2^
Model 1	2.56	0.95	0.91	0.95	0.068	45.76	39		
Model 2	1.17	0.99	0.99	0.99	0.023	99.84	39		
Model 3	3.01	0.95	0.90	0.95	0.077	144.27	48		
Model 3.1	2.24	0.96	0.93	0.96	0.060	76.12	34	-14	-68.25
Model 4	1.30	0.99	0.98	0.99	0.029	64.85	50		
Model 4.1	1.07	1.00	1.00	1.00	0.014	38.44	36	-14	-26.41

*χ^2^, the chi-square, *df*, the degree of freedom, CFI, the comparative fit index, TLI, the Tucker-Lewis coefficient, IFI, Bollen’s incremental fit index, RMSEA, the root mean square error of approximation.*

#### Adolescent Girls

**Figure 3** presents the path details of the model for girls (Model 3.1). Four interaction paths were found to be significant: “Prosocial behavior × relational aggression → peer rejection”; “prosocial behavior × relational aggression → peer attachment”; “prosocial behavior × relational aggression → number of friends”; and “prosocial behavior × relational aggression → relational victimization.” These findings suggested that prosocial behavior might moderate the associations of relational aggression with peer rejection, number of friends, peer attachment, and relational victimization, which supported Hypothesis 2b in part. More specifically, the results of simple slope tests (Dearing and Hamilton, 2006) showed that the relationship between relational aggression and peer rejection among the adolescent girls was significantly positive when levels of prosocial behavior were low (simple slope = 0.90, *t* = 8.44, *p* < 0.01), but non-significant when levels of prosocial behavior were high (simple slope = 0.33, *t* = 2.39, *p* > 0.05). Surprisingly, the findings also showed that the direct association between relational aggression and relational victimization in the adolescent girls was found to be non-significant when levels of prosocial behavior were low (simple slope = -0.24, *t* = -1.62, *p* > 0.05), but it became significantly positive when levels of prosocial behavior were high (simple slope = 0.69, *t* = 3.73, *p* < 0.05). In addition, the relationship between relational aggression and peer attachment among the adolescent girls was found non-significant when levels of prosocial behavior were low (simple slope = 0.12, *t* = 1.48, *p* > 0.05), but it was significantly negative when levels of prosocial behavior were high (simple slope = -0.22, *t* = -2.15, *p* < 0.05). Because “number of friends” was not found to be related to the adolescent girls’ relational victimization, we did not conduct any further analysis on the interaction path “prosocial behavior × relational aggression → number of friends.” Moreover, it is noteworthy that the paths “prosocial behavior → peer attachment” and “overt aggression → peer rejection,” which have been found significant in Model 1, became non-significant when considering the moderator role of prosocial behavior in this model.

#### Adolescent Boys

**Figure 4** presents the path details of the model for boys (Model 4.1). Only one significant interaction path (prosocial behavior × relational aggression → peer rejection) was found in this Model, which suggested that prosocial behavior might have a moderating effect on the relationship between relational aggression and peer rejection for adolescent boys, which also partially supported Hypothesis 2b. The results of a simple slope test showed that the relationship between relational aggression and peer rejection in adolescent boys was significantly positive when the level of prosocial behavior was low (simple slope = 0.90, *t* = 7.00, *p* < 0.01), but became non-significant when the level of prosocial behavior was high (simple slope = 0.25, *t* = 1.92, *p* > 0.05).

## Discussion

In the present study, we testified several models that account for the relations between adolescents’ different types of aggressive behaviors and their experiences of relational victimization by considering the roles of peer relationships, prosocial behavior and gender. Several important findings that have implications for future research and practices have been yielded.

### Peer Rejection as a Mediator

First, we attempted to confirm the results of an earlier study (Ostrov, 2008), based on the theory of Boivin et al. (2001), in which forms of aggression were linked with relational victimization partially mediated by peer rejection in a childhood sample. We extended the study by examining a model based on a sample of Chinese adolescents and by adding friendship into the mediation model. The results suggested that only adolescent girls’ relationally aggressive behaviors could be directly linked with their experiences of relational victimization. The finding, similar to that of Ostrov’s study, partially supports Maccoby’s (1998) two-world hypothesis. According to the hypothesis, relational aggression will predict relational victimization particularly for girls, mainly because of the “gender-segregation” in the peer groups. Such a finding might imply that interventions for relational aggression among girls are especially urgent because it may be an important risk factor for relational victimization.

Consistent with Ostrov’s study as well as other previous studies, we also found that after controlling for the compensatory effect of prosocial behavior, both relationally and overtly aggressive adolescent boys and girls might be more often rejected by their peers, which, in turn, could make them targets of relational aggression. Thus, the indirect pathway is supported in the Chinese culture. That is, as aggressive behaviors might damage the Chinese cultural values that emphasize harmonious interpersonal relationships, Chinese children who are aggressive may be more likely to be rejected by peers, and in turn, may be more likely to be victimized. Therefore, now that peer rejection has been found to play an important role in the relationship between aggression and relational victimization for both adolescent boys and girls, it is crucial for studies to search for important protective factors (e.g., prosocial behavior) that may buffer such a mediating effect to some degree.

### The Roles of Prosocial Behavior

Importantly, we explored the compensatory and moderating effects of prosocial behavior on the direct and indirect pathways from forms of aggression to relational victimization mediated by peer relationships among adolescent girls and boys, respectively. It was initially assumed that prosocial behavior may be a compensatory factor that can directly and indirectly counteract the effects of aggression on relational victimization. Although previous studies have found that prosocial behavior was linked to peer victimization (e.g., Schwartz et al., 1993; Boulton and Smith, 1994; Ostrov et al., 2004), we did not find a direct link between them. Instead, the pathway was indirect via peer relationships (i.e., peer rejection and peer attachment) among both adolescent girls and boys after controlling for the roles of overt and relational aggression. The possible reason that might explain this partial inconsistency with previous studies is that the participants in this study were adolescents instead of younger children, and the peer relationships might have had a greater impact on adolescents than their younger counterparts (Brown, 2004). However, the previous studies were either based on samples of younger children or they seldom considered peer relationships as a mediator when examining the relationship between prosocial behavior and peer victimization. In addition, there might be some cultural differences since the majority of the previous studies were conducted in Western countries. As the Chinese culture emphasizes harmonious interpersonal relationships, individuals with behaviors that promote these values, such as prosocial behavior, may be more favorably evaluated and be less rejected by most of their peers. Hence, additional research based on samples from different cultural backgrounds is needed in the future. Nevertheless, such findings provide evidence in part for a compensatory model of resiliency (Fergus and Zimmerman, 2005) in which prosocial behavior indirectly counteracts the effects of aggression on relational victimization through reducing adolescents’ peer rejection and promoting adolescents’ peer attachment.

In addition, drawing from Hawley’s RCT (Hawley, 2003) as well as the protective model of resiliency (Fergus and Zimmerman, 2005), we then hypothesized that prosocial behavior might also serve as a moderator that buffers the links between aggression, peer relationships, and relational victimization among adolescent girls and boys. Our findings partially support this hypothesis. First, consistent with the protective model of resiliency, we found prosocial behavior buffered the positive effect of relational aggression on peer rejection for both adolescent girls and boys, which might in turn reduce their experiences of relational victimization. More specifically, while relationally aggressive adolescents with low levels of prosocial behaviors might be significantly more likely to be rejected by their peers, those who had more prosocial behaviors might not necessarily be more disliked by their peers, and thus might be less frequently victimized. Such a finding is also in line with Hawley’s RCT, suggesting bistrategic (both aggressive and prosocial) adolescents appear to be effective resource controllers who can achieve and maintain high-status reputations in peer group (e.g., Cillessen and Rose, 2005).

However, surprisingly, our findings also suggest that, for adolescent girls, prosocial behavior might not always be helpful. For instance, although relationally aggressive girls with high levels of prosocial behavior might be less rejected by most peers; at the same time, they might have lower levels of peer attachment and be more likely to experience relational victimization. Due to a single-informant measure of prosocial behavior as well as a cross-sectional nature, we are greatly cautious when explaining this part of the findings. Nevertheless, considering that it might be the case that some victims are targeted by specific peers (for instance, the high-status or the highly prosocial girls) within or out of friendships, but not by everyone, such findings are, to some extent, in line with the group dynamics perspective for explaining aggression in peer groups (see Cillessen and Mayeux, 2007). According to this viewpoint, aggression may occur as an immediate response to situational cues like threats to self. Specifically, if a child’s position of high status is threatened by a peer in some manner (e.g., Using both coercive and prosocial strategies to achieve social dominance status), the child might become aggressive against or deliberately alienate themselves from the competitor. Such an effect may be exacerbated in the peer groups of adolescent girls, because status is a central component of their self concept.

Altogether, these results imply that in general, prosocial behavior may be an important protective factor for relational victimization among both adolescent girls and boys, because it might counteract the negative effects of aggression and buffer the positive effect of relational aggression on peer rejection. However, the moderating effects of prosocial behavior might be more complicated for relationally aggressive girls. Future multi-informant research that distinguishes victims targeted by the most peers from those targeted by specific peers (especially friends) is needed to better understand the roles of prosocial behavior. Such research is significant for both research and practice, because prosocial behavior has been commonly considered as an important strategy that might help decrease peer victimization for both genders in all situations.

### Gender Differences

Moreover, we explored the roles of gender, which yielded several interesting findings. First, adolescent boys reported more experiences of relational victimization than adolescent girls. Although inconsistent with some previous Western studies (e.g., Underwood, 2003; Putallaz et al., 2007; Smith et al., 2010), this finding is similar to the majority of the studies based on Chinese samples (e.g., Ji et al., 2004; Schwartz et al., 2010). In addition, our results showed that there were gender differences in three antecedents: relational aggression, overt aggression, and peer attachment, which are consistent with some studies based on the Eastern and Western samples (e.g., Raja et al., 1992; Jenkins et al., 2002; Archer, 2004; Ji et al., 2004; Kawabata et al., 2010). Altogether, these results are in line with the findings of Schwartz et al. (2001) that in the Chinese cultural context, peer victimization is a more central issue for boys than girls (i.e., boys are initiators and recipients of bullying more often than girls). Importantly, such gender differences in the antecedents might, to some extent, help explain why adolescent boys might have more experiences of relational victimization than girls in the Chinese context. To be specific, when compared to adolescent girls, Chinese boys might have more relationally and overtly aggressive behaviors, which both might be risk factors for relational victimization? At the same time, their relations with friends, which could be a protective factor for relational victimization, are not as good as those of girls. Altogether, these factors might make them easier targets of relational aggression by their peers.

### Limitations

The present study has a number of strengths, such as proposing theoretically driven hypotheses, exploring the mediating effect of peer relationships while considering the roles of prosocial behavior and gender based on a Chinese adolescent sample, and yielding meaningful findings that, to some degree, extend the understanding of relational victimization and its relationships with adolescents’ negative and positive behaviors as well as peer relationships. Conversely, our study has a number of limitations that must be addressed. First, our study had a cross-sectional design, which precluded confirming a causal relationship from aggression, prosocial behavior, and peer relationships to the changing nature of relational victimization over time within and across gender cohorts. Second, although we used various sources of information for different variables accordingly (i.e., peer-nomination reports for aggressive behaviors, prosocial behaviors, and peer rejection; self-reports for the rest of the variables), multi-informant reports for each variable should be recommended in future research to ensure that the measures are sufficiently objective. Third, although we examined the gender difference in adolescents’ relational victimization, there could be gender bias (Bevans et al., 2013) as early as in the measurement of peer victimization, which should be taken into account in future research.

## Conclusion

Despite its limitations, the findings of this study extend our current knowledge regarding relational victimization in several ways. First, in a sample of Chinese adolescents, relational aggression was found to be directly associated with relational victimization for only girls, and peer rejection was found to mediate the relationship between forms of aggression and relational victimization for both girls and boys. Second, a novel contribution of this study is that prosocial behavior was found to play an important role in the relations between aggression, peer relationships and relational victimization when considering the role of gender. Specifically, prosocial behavior might counteract the effect of aggression indirectly by reducing peer rejection and promoting peer attachment; at the same time, it might also buffer the positive association between relational aggression and peer rejection for both adolescent girls and boys. However, the moderating effects of prosocial behavior might be more complicated for relationally aggressive girls. Finally, like most of the studies in China, we found the adolescent boys reported more experiences of relational victimization than girls, probably because they had more relationally and overtly aggressive behaviors whereas having lower friendship quality when compared with girls.

## Conflict of Interest Statement

The authors declare that the research was conducted in the absence of any commercial or financial relationships that could be construed as a potential conflict of interest.
